# Occurrence of adjacent segment fractures after surgical treatment of an osteoporotic vertebral fracture: a retrospective comparison between two different treatment methods

**DOI:** 10.1007/s00402-022-04434-0

**Published:** 2022-04-11

**Authors:** Nazeer Aboud, Niklas Eckardt, Marcel A. Kamp, Christian Senft, Falko Schwarz

**Affiliations:** 1 Department of Neurosurgery, Jena University Hospital, Friedrich Schiller University Jena, Jena, Germany; 2 Department for Radiology, Jena University Hospital, Friedrich Schiller University Jena, Jena, Germany

**Keywords:** Percutaneous surgery, Osteoporotic fracture, Kyphoplasty, Pedicle screw fixation

## Abstract

**Introduction:**

Osteoporotic vertebral fractures are a major healthcare problem. Vertebral cement augmentation (VCA) is frequently used as a minimally invasive surgical approach to manage symptomatic fractures. However, there is a potential risk of adjacent segment fracture (ASF), which may require second surgery. The addition of transcutaneous screw-fixation with cement augmentation superior and inferior to the fracture [Hybrid transcutaneous screw fixation (HTSF)] might represent an alternative treatment option to reduce the incidence of ASF.

**Materials and methods:**

We retrospectively compared surgery time, hospital stay, intraoperative complication rate and the occurrence of ASF with the need for a surgical treatment in a cohort of 165 consecutive patients receiving either VCA or HTSF in our academic neurosurgical department from 2012 to 2020. The median follow-up was 52.3 weeks in the VCA-group and 51.9 in the HTSF-group.

**Results:**

During the study period, 93 patients underwent VCA, and 72 had HTSF. Of all patients, 113 were females (64 VCA; 49 HTSF) and 52 were males (29 VCA; 23 HTSF). The median age was 77 years in both groups. Median surgery time was 32 min in the VCA-group and 81 min in the HTSF-group (*p* < 0.0001). No surgery-related complications occurred in the VCA-group with two in the HTSF-group (*p* = 0.19). ASF was significantly higher in the VCA-group compared to HTSF (24 [26%] vs. 8 [11%] patients; *p* < 0.02). The proportion of patients requiring additional surgery due to ASF was higher in the VCA-group (13 vs. 6%), but this difference was not statistically significant (*p* = 0.18). Median hospital stay was 9 days in the VCA-group and 11.5 days in the HTSF-group (*p* = 0.0001).

**Conclusions:**

Based on this single-center cohort study, HTSF appears to be a safe and effective option for the treatment of osteoporotic vertebral compression fractures. Surgical time and duration of hospital stay were longer in the HTSF-group, but the rate of ASF was significantly reduced with this approach. Further studies are required to ascertain whether HTSF results in superior long-term outcomes or improved quality of life.

## Introduction

Osteoporotic vertebral compression fractures have a high incidence especially with the progress of age and have direct and indirect negative consequences on patient’s health-related quality of life. They are considered a major health care problem worldwide [1–5].

Conservative treatment is only partially effective, and many patients suffer from persistent pain, progressive functional limitation and loss of mobility. In addition, some anti-inflammatory drugs and certain types of analgesics are poorly tolerated by elderly patients. In addition, bed rest leads to further demineralization and may predispose patients to future fractures [6–8].

In 1987, Galibert et al. introduced vertebroplasty (VP) to treat patients with painful vertebral angiomas [9]. The use of vertebroplasty has since been expanded to treat therapy-resistant vertebral compression fractures and to prevent further loss of vertebral body height or progression of a kyphotic deformity [10].

However, this procedure can lead to significant complications [11, 12]. High pressure is often required to inject the low-viscosity cement, which sometimes leads to cement leakage into the spinal canal and peri-spinal vascular system [11, 12].

**Fig. 1:**
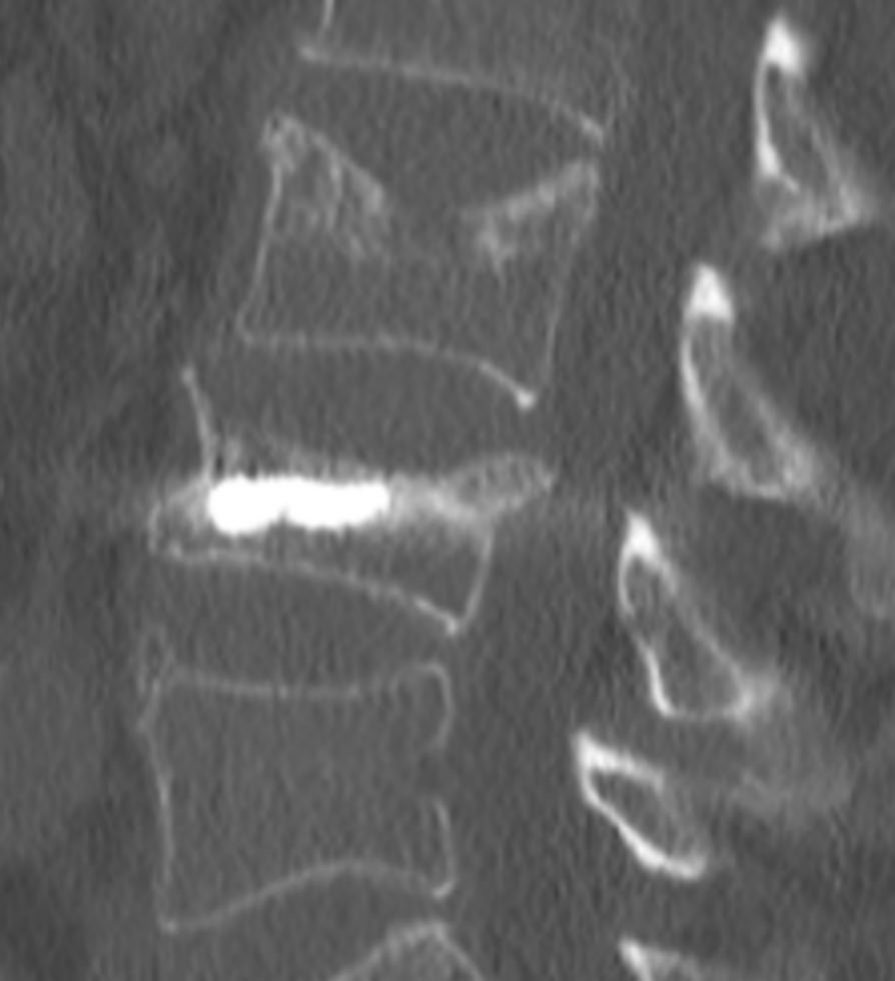
Sagittal CT-Scan showing an ASF after kyphoplasty in the superior vertebral body

As a modification of VP, balloon kyphoplasty (BK) was introduced later [13]. BK reliably reduces intense pain and aims to reconstruct the vertebral body anatomy with correction of the kyphotic deformity. However, many studies [7, 14] reported that the potentiality of adjacent segment fractures (ASF) increases after both means of vertebral cement augmentation (VCA), which may in turn necessitate subsequent surgery (Fig. 1).

Another method to treat vertebral fractures is to combine the transpedicular balloon vertebroplasty with a posterior instrumentation one level above and one level under the index vertebra [15, 16].

**Fig. 2:**
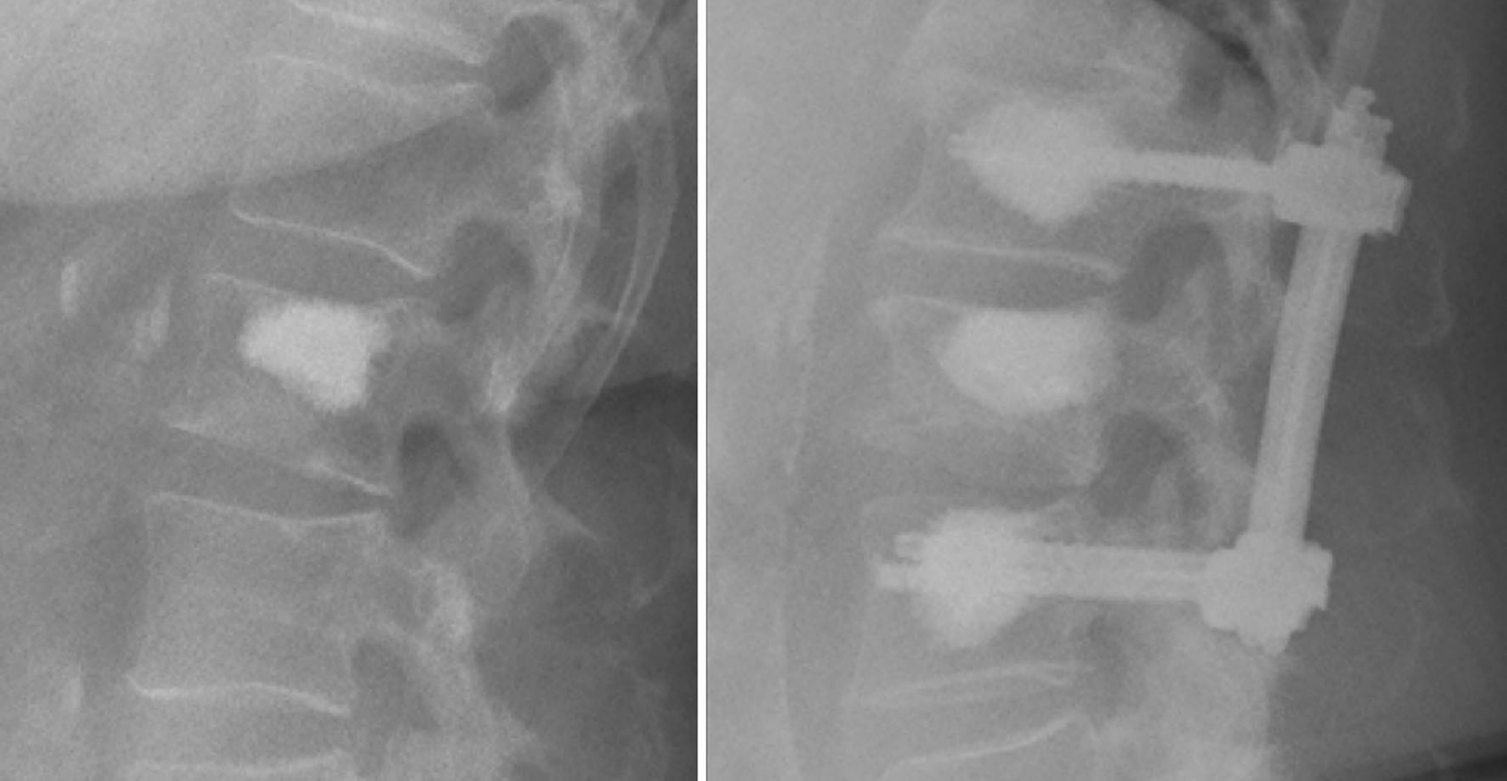
Sagittal X-ray of the lumbar spine. Left: vertebral cement augmentation (VCA); right: transcutaneous cement-augmented screw fixation with vertebral cement augmentation (HTSF)

We hypothesized that by addition of a posterior percutaneous transpedicular instrumentation using cement-augmented cannulated fenestrated screws, we might reduce the incidence of ASF for the patients with vertebral body fractures (Fig. 2), and began with this type of treatment in 2012. In the present study, we analyzed clinical data of patients who had been treated with either conventional VCA or HTSF due to osteoporotic vertebral compression fractures.

## Materials and methods

We retrospectively collected data of all patients who underwent either VCA or HTSF in our institution between January 2012 and December 2020. The median follow-up was 52 weeks.

Following parameters were assessed: patient demographics, fracture characteristics (classification, age), procedural details, intraoperative complications, occurrence of ASF, time to ASF, and revision surgeries caused by ASF.

Inclusion criteria were osteoporotic fractures of one vertebral body (OF types 2 (deformation with no or minor posterior wall involvement) and 3 (deformation with distinct posterior wall involvement)) without neurological deficit, located between Th1 and L5.

Exclusion criteria were OF types 1, 4, and 5, patients with neurological deficits, patients who required additional decompression of the dura, multiple vertebral fractures, or malignancy-associated vertebral fractures.

The primary endpoint was the occurrence of ASF, which was defined as fracture of the first neighboring vertebral body that was newly diagnosed following VCA or HTSF on computed tomography (CT) and/or a magnetic resonance imaging (MRI).

We also compared surgery times, duration of hospital stay, surgical complications and the need for subsequent surgical treatment due to ASF.

The typical follow-up treatment for these patients after surgery was inpatient rehabilitation treatment after hospitalization, followed by outpatient physiotherapy if necessary. A supportive girdle was used only in very rare cases. The posttreatment regimen was similar in both groups.

### Surgical technique

All surgeries were performed in prone position under general anesthesia. Two single-plane mobile C-arms were positioned to confirm the anterior–posterior and lateral views of the fractured vertebra.

VCA was performed as kyphoplasty with the Joline^®^ Spine-Set (Germany). Briefly, a deflated balloon was inserted into the vertebral body through the pedicle and inflated. The balloon then was deflated and removed. The cavity created by the balloon allowed cement injection with minimal pressure, thus minimizing cement leakage.

In the HTSF-group, in addition to the kyphoplasty procedure, a fixation using a posterior percutaneous transpedicular instrumentation using cement-augmented cannulated fenestrated screws was performed [15, 16]. This was also done under fluoroscopic guidance. Screw diameter and screw length were determined based on a preoperative CT scan. Two rods were contoured to pass to the sagittal alignment of the patient and inserted percutaneously to maintain the vertebral body height.

The decision whether VCA or HTSF was performed was based upon the discretion of the surgeon in each individual case.

### Radiological evaluation

Pre- and postoperative X-Ray and low-dose CT scans were obtained in routine fashion. MRI was performed for all patients preoperatively, and for patients who experienced new pain with suspect of an ASF during the follow-up period.

### Statistical analysis

Data were analyzed using IBM^®^ SPSS^®^ statistics software, version 22 for windows. Differences in sex ratio, fracture level ratio and complication ratio between groups were compared using a Chi-square test. Differences in compilation were compared using Fisher Exact Test. Independent data, including age, BMI, and BMD were compared using the Mann–Whitney *U* test. *P* values ≤ 0.05 were considered statistically significant.

## Results

One hundred and sixty-five consecutive patients with osteoporotic fractures were included. Ninety-three patients were treated with VCA and 72 patients with HTSF. Median age in both groups was 77 years (Table 1). Patients in the VCA-group had a median BMI of 26 (IQR 24.2–28.8) and in the HTSF-group 27.2 (IQR 23.7–29.7) (*p* = 0.53). Most fractures occurred in the thoracolumbar junction (T11–L2): 61 (65.6%) patients in VCA-group and 53 (73.6%) patients in HTSF-group. There were no significant differences concerning fracture distribution (*p* = 0.26).

**Table 1:** Clinical data of the VCA and HTSF-group

	VCA				HTSF				*p*
	93				72				
		ASF-VCA	Non-ASF-VCA	*p*		ASF-HTSF	Non-ASF-HTSF	*p*	
Age (years) (IQR: Q1–Q3)	77 (68–81)	75 (60–80)	77 (86–81)	0.88	77 (70–84)	74 (71–82)	77 (70–84)	0.76	0.3
BMI (kg/m^2^) (IQR: Q1–Q3)	26 (24.2–28.8)	25.2 (24.4–29.6)	26 (23.5–28.8)	0.49	27.2 (23.7–29.7)	25.9 (23.5–27. 3)	27 (23.8–31)	0.42	0.53
Fracture level *N* (%)									
T1–T10	8 (8.6%)	4 (16.7%)	4 (5.8%)	0.048	2 (2.8%)	1(12.5%)	1(1.6%)	0.17	0.26
T11–L2	61 (65.6%)	11(45.8%)	50 (72.5%)		53 (73.6%)	6 (75%)	47 (73.4%)		
L3–L5	24 (25.8%)	9 (37.5%)	15 (21.7%)		17 (23.6%)		16 (25%)		
OF classification 2/3 *n* (%)	58/35 (62%/38%)	19/5 (79%/21%)	39/30 (57%/43%)	0.06	30/42 (42%/58%)	4/4 (50%/50%)	26/38 (41%/59%)	0.71	0.008
Follow-up (weeks) (IQR: Q1–Q3)	52.3 (20–154)				51.9 (18–77)				0.12
ASF *n*/(%)	24 (26%)				8 (11%)				0.018
Cranial	10 (42%)				6 (75%)				
Caudal	9 (38%)				2 (25%)				
Cranial and caudal	5 (21%)				0 (0%)				
Time to ASF (weeks) (IQR: Q1–Q3)	9.9 (6.1–45.2)				11.4 (5.4–14.3)				0.4
Sex (F/M)	64/29	17-Jul	47/22	0.8	49/23	07-Jan	42/22	0.21	0.92
Reoperation	12 (13%)				4 (6%)				0.18
Surgery time (minutes) (IQR: Q1–Q3)	32 (28–39)				81 (65–98)				0.00001
Complications	0				2				NA
Hospital stay (days) (IQR: Q1–Q3)	9 (7–13)				11 5 (9–15.25)				0.0002

Values are presented as median (25–75% interquartile range)

*L* lumbar, *Th* thoracic.

In the VCA-group, 35 patients (38%) had an OF type 3 fracture (deformation with distinct posterior wall involvement) and 58 patients (62%) an OF type two fracture (deformation with no or minor posterior wall involvement). In the HTSF-group, 42 patients showed a type 3 (58%) and 30 patients a type 2 (42%) fracture (*p* = 0.008).

Median follow-up was 52.3 weeks (IQR 20–154 weeks) in the VCA-group and 51.9 weeks (IQR 18–77 weeks) in the HTSF-group (*p* = 0.12).

We observed a statistically significantly different occurrence rate of ASF between the two treatment modalities. Twenty-four patients (26%) in the VCA-group and 8 patients (11%) in the HTSF-group developed an ASF during the follow-up period (*p* = 0.018).

Ten (42%) of the ASFs were above and 9 (38%) ASF below the initially operated vertebra in the VCA-group. In five (21%) patients, the ASF occurred both above and below the operated vertebra. In the HTSF-group there were six ASF (75%) above and two (25%) below the treated vertebral body.

The median time until the occurrence of the ASF in our cohort was 10.9 weeks (IQR 6.1–26.9 weeks). In detail, 9.9 weeks (IQR 6.1–45.2 weeks) in the VCA-group and 11.4 weeks (IQR 5.4–14.3 weeks) in the HTSF-group (*p* = 0.4).

Neither patient sex nor age was associated with ASF occurrence. Sixty-four women and 29 men were included in the VCA-group, while in the HTSF-group, there were 49 women and 23 men (*p* = 0.92). The ASF-VCA-group consisted of 17 women and 7 men and the non-ASF-VCA-group of 47 woman and 22 men (*p* = 0.8). There were seven women and one man in the ASF-HTSF-group. In the non-ASF-VCA-group, there were 42 woman and 22 men (*p* = 0.21). Patients with an ASF in the VCA-group were in median 75 years (interquartiles range (IQR): 60–80 years), and in the group without an ASF, 77 years (IQR 68-81 years) (*p* = 0.88). In the HTSF-group, patients with an ASF were 74 years (IQR 71–82 years) and without an ASF, 77 years (IQR 70–84 years) (*p* = 0.76).

As per the secondary endpoints, 12 patients (13%) in the VCA-group, and 4 (6%) patients in the HTSF-group needed to undergo a second surgery due to ASF (*p* = 0.18).

Comparing the surgical techniques, HTSF was more time consuming than VCA. The median duration of surgery in the VCA-group was 32 min (IQR 28–39 min) while it was 81 min in the HTSF-group (IQR 65–98 min) (*p* = 0.00001). Both techniques were comparably safe. No intraoperative complications were documented in the VCA group, whereas two patients had complications during HTSF (hemothorax *n* = 1, and pneumothorax, *n* = 1). One patient in the HTSF-group required surgery due to implant loosening during the follow-up period. There were no statistically significant differences regarding surgical complications between the two treatment groups (*p* = 0.19).

Patients undergoing VCA had a shorter hospital stay than patients with HTSF (median: 9 [IQR 7–13 days] vs. 11.5 [IQR 9–15.3 days] *p* = 0.0002.

## Discussion

Osteoporotic vertebral fractures may lead to refractory back pain, kyphotic deformity, and a reduction in quality of life in the elderly patients [1, 4]. To control these symptoms which are refractory to conservative treatment, the vertebroplasty or kyphoplasty may be used [17].

Transpedicular balloon vertebroplasty for the direct restoration of traumatic burst fractures in combination with posterior instrumentation was used from Verlaan et al. [15] with good results. Many studies have previously reported that the incidence of ASF increases after vertebroplasty or kyphoplasty [12, 18–20], while others have not [21, 22]

Fuentes et al. had used the balloon kyphoplasty (PKP) with additionally percutaneous short-segment pedicle screw osteosynthesis for direct restoration of the vertebral body and showed that could provide a useful approach to patients with traumatic burst vertebral fractures without neurological deficits [16].

The aim of our study was to compare these two methods (VCA and HTSF) since comparative data are lacking.

Here, we observed that the rate of ASF was significantly lower following HTSF than after VCA. Twenty-four patients (26%) in the VCA-group developed an adjacent segment fracture in the follow-up compared to eight patients (11%) in the HTSF-group. However, this superior outcome was at the cost of longer surgical times and a longer hospital stay.

The median time to occurrence of an ASF was similar in both groups and was similar as previously reported [23].

The occurrence of ASF in our study was not related to patient age or sex. This was also comparable with previously published literature [24–30].

There was no significant relationship between the occurrence of ASF and the BMI in our study. Same results were observed by Cao et al. [30]. The other authors found a higher rate of ASF in patients with a higher BMI after kyphoplasty [31]. On contrary, Zhang et al. found that patients with low BMI were at high risk for ASF after vertebroplasty [28].

There was no statistic difference between VCA and HTSF-group in the distribution of the fractures throughout the spine (*p* = 0.26). The most location of the osteoporotic vertebral fractures in each group was in the thoracolumbar spin (T11–L2) followed by lumbar (L3–L5) and thoracic spine (T1–T10).

The most ASFs after surgery occurred in the thoracolumbar region followed by lumbar and thoracic region. This distribution was, compared to patients without ASFs, just in the patients who treated with kyphoplasty statistic important (*p* = 0.048), whereas it was not important in the patients who treated with HTSF (*p* = 0.17) which may mean that the osteoporotic vertebral body fractures in the thoracolumbar region could be better treated with HTSF to reduce the ASF.

The occurrence of ASF implies that patients might require additional therapy, perhaps even a second surgical procedure, which in turn negatively impacts patient quality of life and health care costs. The proportion of patients with ASF who needed to be operated upon was higher following VCA than HTSF, however, not on a statistically significant level.

### Strengths and limitations

The strength of the study is the large sample number with 165 patients.

However, this study has also some limitations. The monocentric study is a purely retrospective analysis without randomization. Apart of the neurological status, the clinical information of the patients including the degree of pain and medications, e.g., long-term use of corticosteroids or immunosuppressants, were not examined. Another aspect that must be mentioned is that the decision to do a VCA or HTSF surgery was dependent on the surgeon. The fact that more patients with OF classification three were treated with HTSF could be due to surgeon bias (perhaps to perform more stabilization). However, in both groups, there was no correlation between OF classification and the occurrence of ASF.

## Conclusion

Based on this single-center cohort, HTSF appears to be a safe and effective option for the treatment of osteoporotic vertebral compression fractures in elderly patients, especially with a fracture in the thoracolumbar region. The duration of surgery and the hospital stay were longer than in the VCA-group, but the rate of ASF can be significantly reduced with the HTSF approach.

Further prospective studies are required to address whether HTSF-patients have a lower revision rate or better postoperative quality of life.
